# From BACE1 Inhibitor to Multifunctionality of Tryptoline and Tryptamine Triazole Derivatives for Alzheimer’s Disease

**DOI:** 10.3390/molecules17078312

**Published:** 2012-07-10

**Authors:** Jutamas Jiaranaikulwanitch, Piyarat Govitrapong, Valery V. Fokin, Opa Vajragupta

**Affiliations:** 1 Department of Pharmaceutical Chemistry, Faculty of Pharmacy, Mahidol University, 447 Sri-Ayudhya Road, Bangkok 10400, Thailand; 2 Center for Neuroscience, Faculty of Science, Mahidol University, 272 Rama VI Road, Rajathevi, Bangkok 10400, Thailand; 3 Department of Chemistry, The Scripps Research Institute, 10500 North Torrey Pines Road, La Jolla, CA 92037, USA

**Keywords:** multifunction drugs, BACE1 inhibitor, anti-amyloid aggregation, chelator, antioxidant, neuroprotection

## Abstract

Efforts to discover new drugs for Alzheimer’s disease emphasizing multiple targets was conducted seeking to inhibit amyloid oligomer formation and to prevent radical formation. The tryptoline and tryptamine cores of BACE1 inhibitors previously identified by virtual screening were modified *in silico* for additional modes of action. These core structures were readily linked to different side chains using 1,2,3-triazole rings as bridges by copper catalyzed azide-alkyne cycloaddition reactions. Three compounds among the sixteen designed compounds exerted multifunctional activities including β-secretase inhibitory action, anti-amyloid aggregation, metal chelating and antioxidant effects at micromolar levels. The neuroprotective effects of the multifunctional compounds **6h**, **12c** and **12h** on Aβ_1-42_ induced neuronal cell death at 1 μM were significantly greater than those of the potent single target compound, BACE1 inhibitor IV and were comparable to curcumin. The observed synergistic effect resulting from the reduction of the Aβ_1-42_ neurotoxicity cascade substantiates the validity of our multifunctional strategy in drug discovery for Alzheimer’s disease.

## 1. Introduction

Alzheimer’s disease (AD) is a common neurodegenerative disorder with a multifactorial etio-pathology involving β-amyloid peptide (Aβ_40_, Aβ_42_) accumulation, iron deregulation, oxidative damage and decreased acetylcholine levels [1,2,3]. β-Amyloid plaque pathogenesis has been a prime target in the search for new drugs for AD etiology treatment. In recent years, it has been evidenced that the Aβ oligomers are more toxic than the deposition of amyloid fibrils or plaques. These Aβ oligomers show a number of toxicity effects including synaptic damage, chondrial dysfunction, glutamate receptor remodeling and alteration of neurogenesis signaling pathways [4]. Therefore, Aβ obstruction and anti-Aβ aggregation are currently the main targets of interest for AD drug development. Aβ peptides are generated from amyloid precursor protein (APP) by β-secretase and γ-secretase cleaving enzymes. An Aβ peptide monomer can aggregate to form oligomers and finally plaques. Inhibition of β-secretase (BACE1), the key enzyme in Aβ peptide generation, and anti-Aβ aggregation are the most attractive targets to prevent Aβ oligomer formation. Metals are also found to play an important role in the pathophysiology of AD by inducing Aβ aggregation and producing harmful reactive oxygen species (ROS). oxidative stress not only leads to metabolic dysfunction and apoptosis of neurons in AD but also enhances BACE1 expression and activity [5,6]. The bound transition metal ions (Cu(I) or Fe(II)) on Aβ oligomers are able to reduce molecular oxygen to hydrogen peroxide resulting in generation of ROS. Thus, metal chelation and radical scavenging are other attractive approaches to reduce neurotoxicity from amyloid aggregation and free radical generation [5,6].

According to the multi-pathogenesis of AD and the failure in clinical trials of many single target drugs, a multi-target-directed-ligand (MTDL) such as memoquin has been examined in current drug discovery. Memoquin exhibited multifunctional properties, acting as AChE inhibitor, free-radical scavenger and inhibitor of Aβ aggregation [3,7]. In the present study, we concentrated on MTDL development to increase drug efficacy for moderation of amyloid β peptide toxicity. Our multifunctional strategy aimed at inhibition of Aβ oligomer formation, moderation of metal levels and prevention of free radical formation, in addition to inhibition of BACE1 to enhance drug efficacy. From this strategy, we have modified our core BACE1 inhibitor structure by adding moieties to exert multifunctional properties in opposition to the AD etiology.

**Figure 1:**
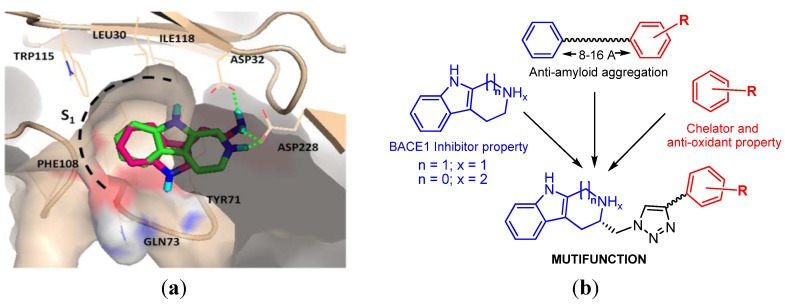
(**a**)The binding mode of the core structures with BACE1 enzyme, the former tryptoline core (green) and new tryptamine core (magenta) and (**b**) the design strategy for multifunctional compounds.

In a previous report, we discovered the core BACE1 inhibitor structure (tryptoline) from virtual screening of Thai medicinal plants [8]. To increase the efficacy, modification of a core structure and multifunctional design were performed. A new core structure (tryptamine) was introduced as a bioisostere of tryptoline in order to increase the hydrogen bond interaction and flexibility. *In silico*, tryptamine showed similar binding as tryptoline. Not only did the indole group of tryptamine fit with the hydrophobic S1 pocket (Leu30, Tyr71, Phe108, and Trp115) but also two hydrogen bonds were formed with catalytic residues Asp32 and Asp228 (Figure 1a). Based on the premise that more hydrogen bonding might yield higher binding affinity, the modification of new tryptamine core was carried out in parallel with the tryptoline core by adding moieties to exert anti-amyloid aggregation, metal chelating and antioxidant effects.

In order to gain the desired effects, an aromatic nucleus substituted with electron donating groups such as hydroxyl and halogen as well as conjugated phenolic moieties was added to the core structures using triazole as a linker (Figure 1b). The addition of aromatic nucleus was projected to produce an anti-Aβ aggregation effect based on the pharmacophore reported by Reinke and Gestwicki [9]. The important anti-Aβ aggregation feature can be achieved with aromatic end groups separated by an optimum length of linker. Moreover, we have introduced active antioxidant and metal chelator functional groups on the added aromatic nucleus [10,11]. The purpose of these moieties was to achieve a multifunctional approach involving anti-Aβ aggregation, metal complexation and radical scavenging action.

## 2. Results and Discussion

### 2.1. Synthesis

**Scheme 1:**
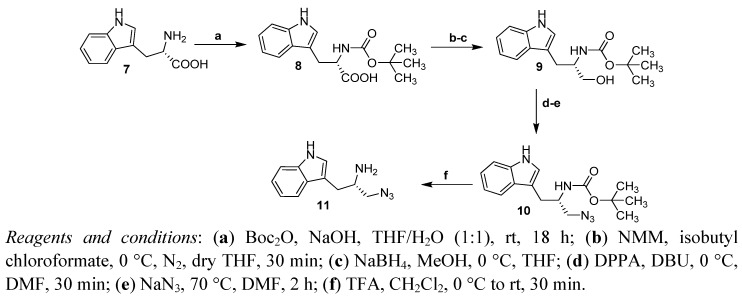
Preparation of *(S)*-3-(-2-amino-3-(1*H*-1,2,3-triazol-1-yl)propyl)indole (**11**).

The tryptoline azide (*S*)-3-(azidomethyl)-2,3,4,9-tetrahydro-1*H*-pyrido[3,4-b]indole (**5**) was synthesized as described previously [8]. The synthetic pathway to the tryptamine core, *(S)*-3-(-2-amino-3-(1*H*-1,2,3-triazol-1-yl)propyl)indole, is shown in Scheme 1.

The amino group of tryptophan (**7**) was protected by a Boc group to yield compound **8** [12]. Then, the carboxylic group of **8** was reduced to hydroxyl with NaBH_4_ [13]. The hydroxyl group of **9** was converted to azide **10** by a substitution reaction with NaN_3_ [14]. Finally, the protecting group was removed to yield tryptamine azide **11** [15].

**Scheme 2:**
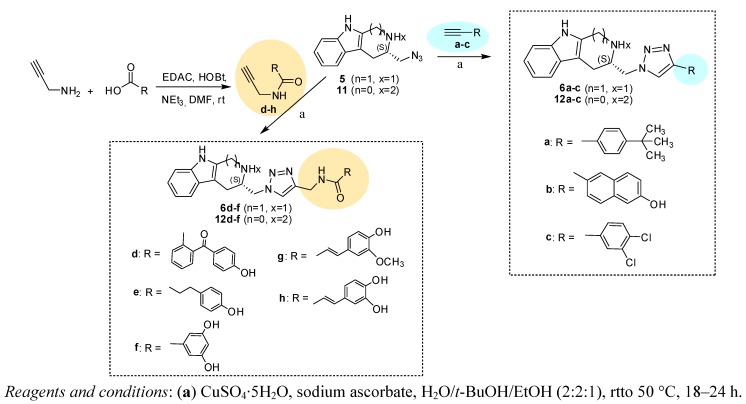
Synthesis of triazolyl methyltryptolines **6a**–**h** and triazolyl methyltryptamines **12a**–**h.**

The azido groups of tryptoline **5** and tryptamine **11** were reacted with different alkynes by copper catalyzed azide-alkyne cycloaddition reactions [16]. Commercial available alkynes **a**–**c** were used in the reaction to afford tryptolines **6a**–**c** and tryptamines **12a**–**c** (Scheme 2). 

Alkynes **d**–**h** were prepared from propargyl amine and carboxylic acid compounds by using EDAC as coupling reagent [17]. The synthesized alkynes were used in the reaction to produce compounds **6d**–**h** and **12d**–**h** (Scheme 2). The chemical and biological properties of synthesized compounds are shown in Table 1. 

### 2.2. Chemical and Biological Assays

**Figure 2:**
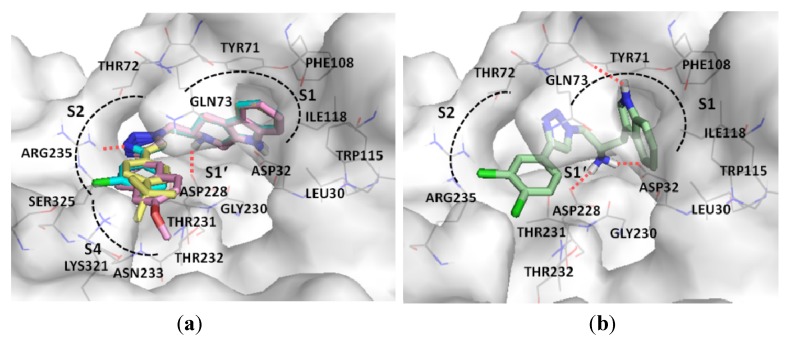
The binding modes of compounds with BACE1 enzyme; (**a**) tryptoline **6a **(yellow), **6b** (pink) and **6c** (cyan); (**b**) tryptamine **12c** (green).

The triazole based compounds of both core structures were evaluated for inhibitory action against BACE1 and for additional activities such as anti-Aβ aggregation, metal chelating and antioxidant (Table 1).The BACE1 inhibitor IV (Calbiochem^®^) was used as a positive control. The BACE1 activity inhibitions were found to be 7.53% and 78.91% at 25 μM. Tryptolines **6a**–**c** and tryptamine **12c** showed good inhibition, with IC_50 _of 18–20 μM, as they all accommodated in the substrate binding site. The binding modes of these four compounds were apparently in a similar manner as shown in Figure 2. The core structures of compounds, tryptoline and tryptamine, fitted with the hydrophobic S1 and S1′ pockets and interacted with residues Leu30, Asp32, Tyr71, Thr72, Gln73, Phe108, Trp115, Ile118, Asp228, Gly230 and Thr231. The NH_2_ group and NH hydrogens in the core structures were involved in hydrogen bonding interactions with Asp32, Asp228 and/or Gln73. The triazole-bearing aromatic side chain accessed the S2-S4 sites and provided interactions with residues Tyr71, Thr72, Gln73, Gly230, Thr231, Thr232, Asn233, Arg235, Lys321 and Ser325 by hydrophobic, dipole induced dipole, dipole-dipole or hydrogen bond interactions. The effect of these compounds against cathepsin-D was determined for selectivity and no inhibition was observed at 100 μM. The interactions of these compounds with the Arg235 residue in S2 possibly contributed to the loss of inhibitory action against cathepsin-D because Asp235 is a unique residue in BACE1 compared with cat-D (Val233) and rennin (Ser222) [18].

**Table 1:** Multifunctionality of triazole based compounds.

Cpd	Log P	BACE1		Anti-Aβ aggregation		Fe chelation		DPPH	
		% Inhibition (at 25 µM) (±SEM)	IC_50 _(µM)	% Inhibition (at 100 µM) (±SEM)	IC_50 _(µM)	% Capacity (at 100 µM) (±SEM)	Stoichio-metric ratio (Fe:cpd)	% Inhibition (at 100 µM) (±SEM)	IC_50 _(µM)
**6a**	4.69	67.95 (±0.35)	19.82	NA	-	9.67 (±0.32)	-	NA	-
**6b ^a^**	3.86	73.63 (±1.47)	18.86	7.19 (±1.19)	-	12.35 (±0.68)	-	NA	-
**6c**	4.10	78.91 (±2.83)	18.03	10.12 (±0.86)	-	15.88 (±0.21)	-	NA	-
**6d**	3.01	14.10 (±1.79)	-	NA	-	32.86 (±0.19)	-	2.81 (±0.37)	-
**6e**	2.16	12.57 (±1.10)	-	NA	-	20.63 (±0.35)	-	5.45 (±0.33)	-
**6f**	1.41	21.28 (±1.92)	-	NA	-	5.80 (±0.04)	-	NA	-
**6g**	2.01	21.14 (±1.13)	-	66.36 (±2.02)	82.90	8.88 (±0.25)	-	47.03 (±0.12)	106.41
**6h**	1.75	21.72 (±0.33)	-	84.13 (±2.49)	29.86	42.74 (±0.30)	1:3	92.29 (±0.02)	42.91
**12a**	4.42	18.91 (±0.40)	-	17.81 (±0.86)	-	42.65 (±1.07)	-	NA	-
**12b**	3.58	30.42 (±3.54)	-	34.02 (±10.13)	-	10.54 (±0.22)	-	NA	-
**12c**	3.83	61.46 (±1.83)	20.75	67.56 (±0.72)	83.23	60.90 (±0.51)	1:3	NA	-
**12d**	2.73	32.97 (±0.37)	-	82.52 (±1.26)	47.51	13.87 (±0.59)	-	1.56 (±0.51)	-
**12e**	1.88	7.53 (±1.79)	-	36.03 (±1.92)	-	41.42 (±1.13)	-	NA	-
**12f**	1.13	16.57 (±2.91)	-	3.80 (±1.56)	-	13.06 (±0.16)	-	1.32 (±0.78)	-
**12g**	1.74	16.23 (±0.45)	-	46.06 (±1.63)	109.83	77.70 (±0.67)	1:3	40.79 (±0.31)	130.44
**12h**	1.47	40.03 (±0.95)	-	81.48 (±2.54)	56.39	66.45 (±0.37)	1:3	50.58 (±0.17)	92.70
Inh IV (Merck^®^)	1.23	96.51 (±1.33)	0.015 ^b^	-	-	-	-	-	-
Curcumin	2.56	-	-	82.90 (±0.82)	0.63^c^	-	-	-	-
EDTA	−2.69	-	-	-	-	98.00 (±0.34)		-	-
Ascorbic acid	−3.36	-	-	-	-	-	-	53.64 (±0.11)	94.92

NA = no activity, previously synthesized compounds [8]; ^b^ Ref. [19]; ^c^ Ref. [20].

**Figure 3:**
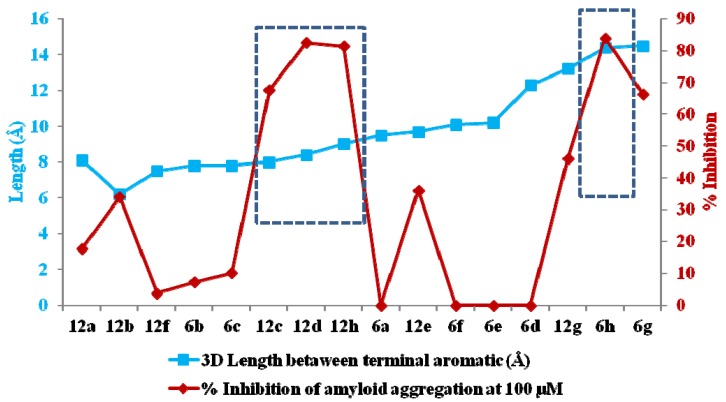
The relationship of bond length between aromatic terminal in the 3D structure and anti-amyloid aggregation activity.

The anti-Aβ aggregation activity of compounds bearing the new tryptamine core was generally higher than those of corresponding compounds with the former tryptoline core with the exception of **12h** (Table 1). The distance between aromatic terminals in the 3D structure after energy minimization was determined to define the relationship between structure and anti-amyloid aggregation activity. Compounds having a length between aromatic terminals of 8–9 Å (compounds **12c**, **12d** and **12h**) and 13–14 Å (compounds **6h** and **6g**) showed anti-Aβ aggregation activity over 50% (Figure 3). The optimal length between aromatic terminals of 8–9 Å is in agreement with Reinke and Gestwicki criteria [9], we also found the new optimal length of 13–14 Å.

**Figure 4:**
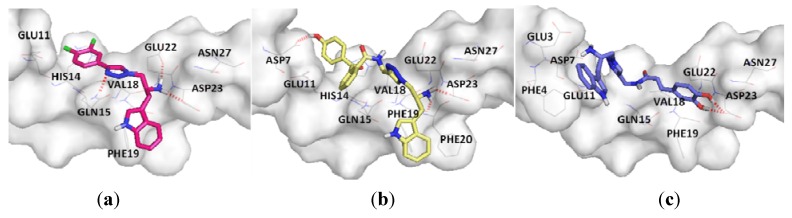
The binding mode of compounds with amyloid-β (1-42); (**a**) **12c** (pink); (**b**) **12d** (yellow); (**c**) **12h** (blue).

The IC_50_ values for anti-Aβ aggregation of tryptolines **6g** and **6h** were 82.90 μM and 29.86 μM, respectively while those of tryptamines **12c**, **12d** and **12h** were 83.23 μM, 47.51 μM, 56.39 μM, respectively. The substantial activity of these compounds possibly resulted from the capability to wrap the Aβ motif which is crucial for aggregation. The formation of H-bonds or hydrophobic interactions of the core structures such as those of tryptamines **12c**, **12d** and **12h** with the key residue Asp23 possibly prevented Aβ from self-aggregation as Asp23 plays an important role in intermolecular salt bridge formation of Aβ peptides in protein aggregation (Figure 4a–c).

**Figure 5:**
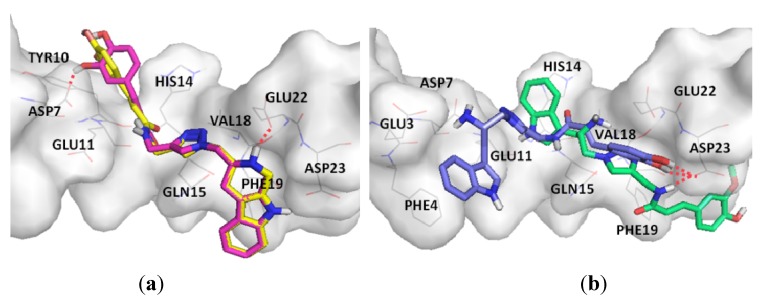
The binding modes of compounds with amyloid-β (1-42); (**a**) **6g** (yellow) and **6h** (pink); (**b**) **12g** (green) and **12h** (blue).

Moreover, apart from the tryptamine and tryptotoline cores, the added moieties **a**–**h** also enhanced the Aβ binding. The *m*-OH substitution on the added aromatic nucleus of **6h** provided a H-bond interaction with Asp7 in addition to the H-bond at Asp23. The interactions at both terminals secured the triazole linker to form hydrophobic and dipole-dipole interactions with Gln15, Val18 and Phe19 residues, these amino acid residues are self-recognition residues in the aggregation process (Figure 5a). Thus, **6h** was two times more potent than **6g**, which provided an H-bond only at one terminal (IC_50_ 29.86 μM *vs.* 82.90 μM), the interactions at the terminal ends appeared to strengthen the Aβ wrapping. In case of **12g** *vs.* **12h** (Figure 5b), tryptamine **12h** provided H-bond interactions at the terminal ends in the same scenario as **6h** but flipped vertically, and the IC_50_ value of **12h** is two times better than that of **12g** (56.39 μM *vs.* 109.89 μM).

**Figure 6:**
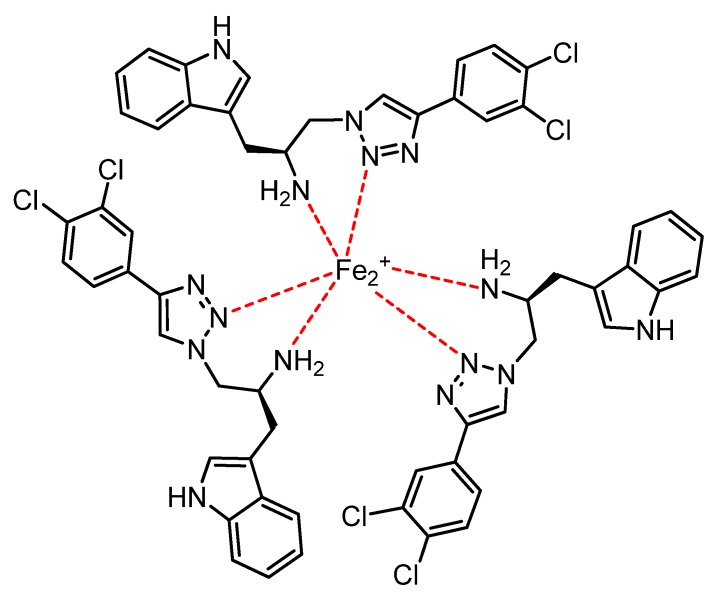
The chelating model of compound **12c **with Fe^2+^.

In metal chelating capability, the tryptoline and tryptamine derivatives had chelating capacity between 5.80–77.70% at 100 µM. Generally, compounds containing the tryptoline core formed complexes with Fe^2+^ with less capacity than those with the tryptamine core due to the restriction ability of the NH in the tryptoline core to chelate with metal. The lone pair of electrons on the nitrogen atom in the core structure as well as the nitrogen atom in the triazole ring were the chelating functions. Compounds **12c**, **12g** and **12h** exhibiting chelating capacities higher than 50% at 100 μM were selected for the determination of stoichiometric ratio. The stoichiometric ratio of these compounds **12c**, **12g** and **12h** per metal were 3:1 (Figure 6).

For free radical scavenging activity, compounds containing conjugated phenolic moieties showed good activity, as anticipated. Compound **6h** had high activity, with an IC_50_ value of 42.91 μM while compounds **6g**, **12g** and **12h** showed moderate antioxidant properties, with IC_50_ values of 106.41 μM, 130.44 μM and 92.70 μM, respectively. Moreover, di-substitution of hydroxyl groups at *m-* and *p-*position helped to enhance the antioxidant activity as in **6h** and **12h** compared with **6g** and **12g**.

**Figure 7:**
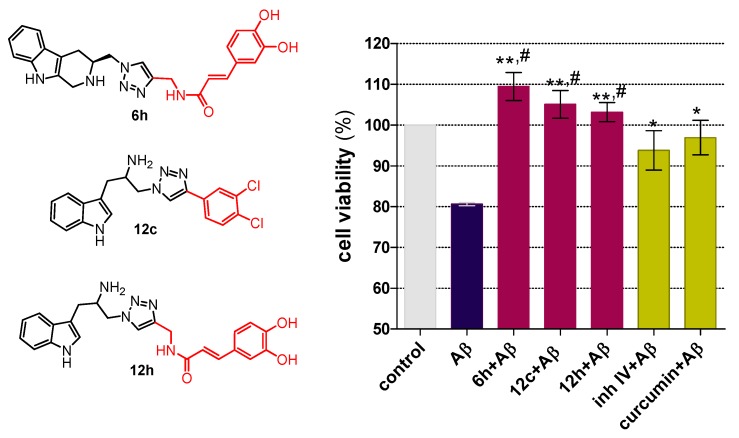
The protective effect of multifunctional compounds against Aβ-induced neuronal cell death by MTT viability assay at 1 μM, *t*-test * *p* < 0.05, ** *p *< 0.01 *vs.* Aβ treated cells and ^#^ *p* < 0.05 *vs.* BACE1 inhibitor IV.

Compounds **6h**, **12c** and **12h** were found to possess multifunctional activity as shown in Table 1. Compound **12c** demonstrated substantial anti-Aβ aggregation and chelating effect in addition to the BACE1 inhibitory action (IC_50_ 20.75 μM). The major effects of compounds **6h** and **12h** were anti-Aβ aggregation and antioxidant activity resulting from the conjugated phenolic side chain while the BACE1 inhibitory action appeared to be moderate. Their IC_50_ values for anti-Aβ aggregation were 29.86 μM and 56.39 μM, and those of antioxidant activity were 42.91 μM and 92.70 μM, respectively. The chelating capabilities of these three compounds were found to be moderate, 40–70% at 100 μM. Compounds **6h**, **12c** and **12h** were selected for further investigation to demonstrate the synergistic effect on neuroprotection against β-amyloid toxicity in SH-SY5Y cells. The effect of these compounds on neurotoxicity induced by Aβ_1-42_ was determined at a non-toxic concentration (1 μM) using a colorimetric MTT method [21]. Curcumin, which is a potent antioxidant, anti-Aβ aggregation and chelating agent was included in the assay together with BACE1 Inhibitor IV. All test compounds significantly inhibited neuronal death induced by Aβ_1-42_ (Figure 7). As anticipated, the neuroprotective effect of the designed compounds using multi-target approach was superior to the single target compound. The multifunctional compounds **6h**, **12c** and **12h** were able to decrease cell death to a greater extent than BACE1 inhibitor IV which is a potent single target compound. BACE1 inhibitor IV also improved cell viability as it reduces the amyloid beta level produced by the increase of BACE1 expression and activity under oxidative stress condition. The activation of the PKR (double-stranded RNA dependant protein kinase) pathway and eIF2α (eukaryotic translation initiation factor-2α) translational control [22] as well as a transcriptional regulation mediated by c-jun N-terminal kinase (JNK) pathway [23,24,25] are the causes of oxidative stress-induced BACE1 elevation. Increased levels of PKR mRNA, PKR protein and BACE1 were observed in SH-SY5Y after oxidative exposure [22]. Thus, inhibition of BACE1 enzyme helps in reducing the production of new amyloid beta peptide that causes neurotoxicity. Although the potency of multi-target compounds was apparently low in THE micromolar level against each individual target, the neuroprotection in SH-SY5Y cells was comparable to that provided by curcumin. The inclusion of BACE1 inhibitory action synergistically enhanced the neuroprotective effects of compounds **6h**, **12c** and **12h** to the same level as curcumin, a potent nanomolar multi-target compound.

## 3. Experimental

### 3.1. General

All ligands were generated and optimized with ChemDraw Ultra 9.0 and Chem3D Ultra 9.0. AutoDock program suit version 4.2on Garibaldi platform at The Scripps Research Institute was employed to perform the docking calculation. All chemical reagents were purchased from Aldrich or AK Science. ^1^H-NMR and ^13^C-NMR spectra were acquired on Bruker Avance 300 or 400 MHz instruments. Mass spectra were recorded on either a Thermo Finnigan or LCMS Bruker MicroTof. IR spectra were recorded on Nicolet FTIR 550. BACE1 enzyme and BACE1 substrate were purchased from Sino Biological®and Calbiochem®, respectively. Amyloid-β (1–42) from Anaspec® was used in ThT and MTT assay.

### 3.2. Docking Study of β-Secretase (BACE1)

The BACE1 template 2IRZ-F was constructed from two crystal structures of β-secretase (BACE1) bound to inhibitors (Protein Data Bank code: 2IRZ [26] and 1FKN [27]) as previously described [8]. Docking parameters in the docking studies were as follows: the number of genetic algorithm (GA) runs was 100; the population size was 150; the maximum number of energy evaluations was increased to 15,000,000 per run; and the maximum number of generations was 27,000.

### 3.3. Docking Study of Amyloid β (Aβ)

Amyloid β peptide (residues 1-42) template was prepared from crystal structure of Aβ monomer (PDB entry code: 1Z0Q [28]). The dimensions of grid were centered on the coordinates −1.733, 3.591 and −6.759 with 120 × 80 × 80 Å and 0.5 Å spacing between grids points. The docking parameters were as follows: the number of GA runs was 100; the population size was 150; the maximum number of energy evaluations was increased to 5,000,000 per run; and the maximum number of generations was 27,000.

### 3.4. Preparation of Azidomethyl Tryptamine Intermediates

*(S)-2-(tert-Butoxycarbonylamino)-3-(1H-indol-3-yl)propanoic acid *(**8**). L-Tryptophan (20.45 g, 0.10 mol) in THF/H_2_O (1:1, 100 mL) was added with sodium hydroxide (8.80 g, 0.22 mol) and di-*tert*-butyl dicarbonate (24.01 g, 0.11 mol). The reaction was stirred at room temperature for 18 h. After the reaction was complete, water was added to dissolve the precipitate, then the THF was removed under reduced pressure and the aqueous layer was extracted with dichloromethane. The aqueous layer was acidified by 1 N HCl to pH 4 and extracted with dichloromethane and ethyl acetate (30 mL × 3). The organic phase was dried over sodium sulphate and concentrated to yield compound **8** as a white powder (24.74 g, 81%); m.p.: 141–143 °C; FTIR (KBr): 3373, 3338, 2524, 1717, 1647, 1505, 1400, 1251, 1163 cm^−1^; ^1^H-NMR (400 MHz, DMSO-*d*_6_): δ 10.82 (s, 1H, H1), 7.50 (d, *J* = 8.00 Hz, 1H, H4), 7.32 (d, *J* = 8.00 Hz, 1H, H7), 7.13 (d, *J* = 1.60 Hz, 1H, H2), 7.05 (t, *J* = 7.40 Hz, 1H, H6), 6.96 (t, *J* = 8.60 Hz, 1H, H5), 4.15–4.10 (m, 1H, H9), 3.11 (dd, *J* = 14.80, 4.80 Hz, 1H, H8_b_), 2.95 (dd, *J* = 14.40, 9.20 Hz, 1H, H8_a_), 1.31 (s, 9H, CH_3_); LRMS (API-ES) *m/z* 631.3 [2M+Na], 327.3 [M+Na].

*(S)-tert-Butyl-1-hydroxy-3-(1H-indol-3-yl)propan-2-yl carba-mate *(**9**). To compound **8** (4.69 g, 15.42 mmol) in dry THF (30 mL) was added *N*-methylmorpholine (2.0 mL, 18.50 mmol) and isobutyl chloroformate (2.4 mL, 18.50 mmol) at 0 °C under nitrogen and the mixture was stirred for 30 min. The precipitate formed was immediately filtered and to the filtrate was added sodium borohydride (1.17 g, 30.84 mmol) at 0 °C. After 15 min, methanol (5 mL) was added dropwise at 0 °C. After the reaction was complete, THF was evaporated off and the residue was diluted with ethyl acetate (30 mL). The organic phase was washed with saturated sodium bicarbonate, 5% potassium bisulphate, and brine and dried over sodium sulphate. The concentrated residue was purified by column chromatography (hex/EtOAc; 7:3) to give a white powder of compound **9** (2.15 g, 48%); m.p.: 122–123 °C; FTIR (KBr): 3420, 3404, 3360, 1686, 1526, 1368, 1247, 1173, 999 cm^−1^; ^1^H-NMR (300 MHz, CDCl_3_): δ 8.13 (s, 1H, H1), 7.63 (d, *J* = 7.81 Hz, 1H, H4), 7.34 (d, *J* = 8.03 Hz, 1H, H7), 7.18 (t, *J* = 7.52 Hz, 1H, H6), 7.11 (t, *J* = 7.41 Hz, 1H, H5), 7.02 (d, *J* = 2.19 Hz, 1H, H2), 4.81 (br, 1H, carbamate NH), 4.02–3.92 (m, 1H, H9), 3.70–3.54 (m, 2H, H10), 2.97 (d, *J* = 6.84 Hz, 2H, H8), 2.52 (s, 1H, OH), 1.40 (s, 9H, CH_3_); LRMS (API-ES) *m/z* 603.3 [2M+Na], 313.2 [M+Na].

*(S)-tert-Butyl-1-azido-3-(1H-indol-3-yl)propan-2-ylcarbamate *(**10**). Under nitrogen, diphenyl-phosphoryl azide (1.2 mL, 5.36 mmol) and DBU (0.8 mL, 5.36 mmol) were added dropwise to a cooled (0 °C) solution of compound **9** (1.04 g, 3.57 mmol) in DMF (5 mL). After the reaction was complete, sodium azide (1.16 g, 17.85 mmol) was added to the reaction at 0 °C and the reaction temperature was raised to 80 °C. The reaction was diluted with ethyl acetate (30 mL) and washed with water (30 mL) twice. The aqueous layer was washed with ethyl acetate (30 mL). Then the organic phases were combined, washed with saturated sodium bicarbonate, brine and dried over sodium sulphate. The concentrated residue was purified by column chromatography (hex/EtOAc; 9:1) to yield a white powder of compound **10** (0.5641 g, 50%); m.p.: 98–99 °C; FTIR (KBr): 3418, 3399, 3354, 2110, 1690, 1518, 1170 cm^−1^; ^1^H-NMR (300 MHz, CDCl_3_): δ 8.07 (s, 1H, H1), 7.62 (d, *J* = 7.63 Hz, 1H, H4), 7.34 (d, *J* = 7.87 Hz, 1H, H7), 7.18 (t, *J* = 7.52 Hz, 1H, H6), 7.11 (t, *J* = 7.41 Hz, 1H, H5), 7.02 (d, *J* = 2.21 Hz, 1H, H2), 4.69–4.67 (br, 1H, NH), 4.06–4.05 (br, 1H, H9), 3.41–3.28 (m, 2H, H10), 3.04–2.88 (m, 2H, H4), 1.40 (s, 9H, CH_3_); LRMS (API-ES) *m/z* 338.3 [M+Na].

*(S)-1-Azido-3-(1H-indol-3-yl)propan-2-amine *(**11**). To compound **10** (0.52 g, 1.65 mmol) in dichloromethane (10 mL) was treated dropwise with trifluoroacetic acid (2.50 mL, 33.04 mmol) at 0 °C and stirred at room temperature for 30 min. Saturated sodium bicarbonate was added to the reaction for adjust pH = 8. The resulting solution was extracted with ethyl acetate 30 mL. The organic phase was dried over sodium sulphate. The concentrated residue was purified with column chromatography (CHCl_3_/MeOH; 20:1) to yield **11 **as a brown oil (0.14 g, 40%): FTIR (ATR): 3407, 3345, 3283, 2097, 1660, 1577, 1091cm^−1^; ^1^H-NMR (300 MHz, DMSO-*d*_6_): δ 10.84 (s, 1H, H1), 7.51 (d, *J* = 7.78 Hz, 1H, H4), 7.33 (d, *J* = 8.03 Hz, 1H, H7), 7.15 (s, 1H, H2), 7.05 (t, *J* = 7.08 Hz, 1H, H6), 6.96 (t, *J* = 7.05 Hz, 1H, H5), 3.27–3.24 (m, 1H, H10_b_), 3.17–3.12 (m, 2H, H10_a_, H9), 2.77 (dd, *J* = 14,10, 5.47 Hz, 1H, H8_b_), 2.65 (dd, *J* = 14.20, 6.51 Hz, 1H, H8_a_); LRMS (API-ES) *m/z* 216.3 [M+H].

### 3.5. Preparation of Alkynes ***d–h***

A mixture of carboxylic acid (2 mmol) and *N*-hydroxybenzotriazole (1 mmol) were dissolved in DMF (5 mL). Propargylamine (2 mmol) and triethylamine (2 mmol) were added to the reaction and stirred at room temperature for 5 min. Then 1-(3-dimethylaminopropyl)-3-ethylcarbodiimide·HCl (EDAC, 2.4 mmol) was added to the reaction. After 18 h, water (20 mL) was added to stop the reaction. The aqueous solution was extracted with ethyl acetate (10 mL × 3). The resulting solution was washed with 1N HCl, saturated NaHCO_3_, brine, dried with anhydrous NaSO_4_ and concentrated. 

*2-(4-Hydroxybenzoyl)-N-(prop-2-ynyl)benzamide *(**d**). Compound **d** was obtained from 2-(4-hydroxybenzoyl) benzoic acid as described above. The concentrated residue was washed with diethyl ether to yield a white powder (0.4239 g, 76%); m.p.: 165–167 °C; FTIR (KBr): 3319, 3308, 3240, 3240, 1654, 1612, 1518, 1346, 1201, 1166 cm^−1^; ^1^H-NMR (300 MHz, DMSO-*d*_6_): δ 9.48 (s, 1H, OH), 7.70 (d, *J* = 6.78 Hz, 1H, H6′), 7.57–7.46 (m, 2H, H4′, H5′), 7.23 (d, *J* = 7.4 Hz, 1H, H3′), 7.14 (d, *J* = 8.66 Hz, 2H, H2″, H6″), 7.01 (s, 1H, NH-amide), 6.69 (d, *J* = 8.70 Hz, 2H, H3″, H5″), 3.99 (dd, *J* = 17.75, 2.41 Hz, 1H, H1_b_), 3.83 (dd, *J* = 17.72, 2.42 Hz, 1H, H1_a_), 2.88–2.86 (m, 1H, H3); LRMS (API-ES) *m/z* 581.2 [2M+Na], 302.2 [M+Na].

*3-(4-Hydroxyphenyl)-N-(prop-2-ynyl)propanamide* (**e**). Compound **e** was obtained from 3-(4-hydro xyphenyl) propinoic acid as described above as a white powder (0.2784 g, 69%); m.p.: 102–104 °C; FTIR (KBr): 3332, 3272, 3186, 1656, 1543, 1518, 1356, 1229, 1172 cm^−1^; ^1^H-NMR (300 MHz, DMSO-*d*_6_): δ 9.12 (s, 1H, OH), 8.24 (t, *J* = 5.24 Hz, 1H, NH), 6.96 (d, *J* = 8.34 Hz, 2H, H2″, H6″), 6.63 (d, *J* = 8.35 Hz, 2H, H3″, H5″), 3.84–3.81 (m, 2H, H1), 3.07 (t, *J* = 2.33 Hz, 1H, H3), 2.67 (t, *J* = 7.79 Hz, 2H, H2′), 2.30 (t, *J* = 7.81 Hz, 2H, H3′); LRMS (API-ES) *m/z* 429.3 [2M+Na], 226.2 [M+Na], 204.3 [M+H].

*3,5-Dihydroxy-N-(prop-2-ynyl)benzamide* (**f**). Compound **f** was obtained from 3,5-dihydroxybenzoic acid as described above. The concentrated residue was washed with diethyl ether to yield a light brown powder (0.3242 g, 85%); m.p.: 82–83 °C; FTIR (KBr): 3518, 3397, 3345, 3271, 1624, 1591, 1524, 1371, 1214, 1160 cm^−1^; ^1^H-NMR (300 MHz, DMSO-*d*_6_): δ 9.49 (s, 2H, OH), 8.66 (t, *J* = 5.52 Hz, 1H, NH), 6.66 (d, *J* = 2.09 Hz, 2H, H2′, H6′), 6.35 (t, *J* = 2.05 Hz, 1H, H4′), 3.97–3.95 (m, 2H, H1), 3.05 (s, 1H, H3); LRMS (API-ES) *m/z* 405.3 [2M+Na], 214.2 [M+Na], 192.3 [M+H].

*(E)-3-(4-Hydroxy-3-methoxyphenyl)-N-(prop-2-ynyl)acrylamide*(**g**). Compound **g** was obtained from ferulic acid as described above. The concentrated residue was purified by column chromatography on silica gel (CHCl_3_/EtOAc 7:3). A colorless oil was obtained (0.2295 g, 50%); FTIR (ATR): 3284, 2115, 1655, 1582, 1511, 1382, 1261, 1202, 1119, 1028 cm^−^^1^; ^1^H-NMR (400 MHz, CDCl_3_): δ 7.53 (d, *J* = 15.56 Hz, 1H, H3′), 6.99 (d, *J* = 7.96 Hz, 1H, H6″), 6.91 (s, 1H, H2″), 6.84 (d, *J* = 8.14 Hz, 1H, H5″), 6.36 (s, 2H, OH, NH), 6.29 (d, *J* = 15.54 Hz, 1H, H2′), 4.14 (s, 2H, H1), 3.81 (s, 3H, CH_3_); LRMS (ESI) *m/z* 484.63 [2M+Na], 254.14 [M+Na], 232.19 [M+H].

*(E)-3-(3*,*4-Dihydroxyphenyl)-N-(prop-2-ynyl)acrylamide* (**h**). Compound **h** was obtained from caffeic acid as described above. The concentrated residue was purified by column chromatography on silica gel (CHCl_3_/EtOH 10:0.5). A white powder was obtained (0.0880 g, 20%); m.p.: 169–170 °C; FTIR (KBr): 3477, 3366, 3266, 1646, 1587, 1536, 1343, 1264, 1191, 973 cm^−1^; ^1^H-NMR (300 MHz, CD_3_OD-d_4_): δ 7.40 (d, *J* = 15.7 Hz, 1H, H3′), 7.00 (d, *J* = 2.02 Hz, 1H, H2″), 6.90 (dd, *J* = 8.21, 2.01 Hz, 1H, H6″), 6.75 (d, *J* = 8.16 Hz, 1H, H5″), 6.34 (d, *J* = 15.68 Hz, 1H, H2′), 4.05 (d, *J* = 2.54 Hz, 2H, H1), 2.59 (t, *J* = 2.54 Hz, 1H, H3); LRMS (ESI) *m/z* 240.06 [M+Na].

### 3.6. Preparation of Triazolylmethyl Tryptolines ***6a–i*** and Triazolylmethyl Tryptamines ***12a–h***

A mixture of compound **5** or **11** (1.0 mmol), alkynes **a**–**i** (1.2 mmol), 5% mol CuSO_4_ and 20% mol sodium ascorbate in *t*-BuOH/H_2_O/EtOH (2:2:1, 10 mL) was stirred at room temperature to 50 °C for 24 h. After ethanol was evaporated out, water (10 mL) was added to the reaction. The aqueous solution was extracted with ethyl acetate (10 mL × 3). The organic solution was washed with brine, dried, concentrated. 

*(S)-3-((4-(4-tert-Butylphenyl)-1H-1,2,3-triazol-1-yl)methyl)-2,3,4,9-tetrahydro-1H-pyrido[3,4-b] indole* (**6a**). Compound **6a** was obtained from compound **5** and 1-*tert*-butyl-4-ethynylbenzene (**a**) as described above. The concentrated residue was purified by column chromatography (CHCl_3_/ EtOAc/NH_4_OH; 5:5:0.1). A light yellow powder of compound **6a** was obtained (0.1261 g, 32%); m.p.: 218–220 °C; FTIR (KBr): 3407, 3293, 1625, 1492, 1226cm^−^^1^; ^1^H-NMR (300 MHz, acetone-*d*_6_): δ 9.84 (s, 1H, H9), 8.40 (s, 1H, H5′), 7.83 (d, *J* = 8.50 Hz, 2H, H2″, H6″), 7.47 (d, *J* = 8.51 Hz, 2H, H3″, H5″), 7.38 (d, *J* = 7.62 Hz, 1H, H5), 7.30 (d, *J* = 7.65 Hz, 1H, H8), 7.05–6.93 (m, 2H, H7, H6), 4.67 (dd, *J* = 13.81, 5.26 Hz, 1H, H10_b_), 4.58 (dd, *J* = 13.83, 7.82 Hz, 1H, H10_a_), 4.06 (d, *J* = 16.34 Hz, 1H, H1_b_), 3.98 (d, *J* = 16.34 Hz, 1H, H1_a_), 3.52–3.43 (m, 1H, H3), 2.70 (dd, *J* = 15.16, 3.20 Hz, 1H, H4_b_), 2.52 (dd, *J* = 15.11, 9.80 Hz, 1H, H4_a_), 1.33 (s, 9H, CH_3_); ^13^C-NMR (300 MHz, acetone-*d*_6_): δ 151.394, 147.682, 137.343, 134.567, 129.701, 128.526, 126.420, 126.030, 121.882, 121.565, 119.457, 118.112, 111.647, 107.588, 55.343, 55.034, 43.211, 35.120, 31.575, 26.611; LRMS (API-ES) *m/z* 793.4 [2M+Na], 771.5 [2M+H], 408.4 [M+Na], 386.4 203.2 [M+H]; HRMS (ESI) *m/z* calcd for [M^+^] 385.5047, Found 408.1892 [M+Na], 386.2073 [M+H].

*(S)-3-((4-(6-Methoxynaphthalen-2-yl)-1H-1,2,3-triazol-1-yl)me-thyl)-2,3,4,9-tetrahydro-1H-pyrido- [3,4-b]indole* (**6b**). Compound **6b** was obtained from compound **5** and 2-ethynyl-6-methoxynaphthalene (**b**) as described above. After water was added to the reaction mixture, the precipitate was filtered through sintered glass and washed with water and ethyl acetate to yield a light brown solid of **6b** (0.1548 g, 38%); m.p.: 232–234 °C; FTIR (KBr): 3421, 3302, 3243, 1613, 1501, 1266, 1215, 1029 cm^−1^; ^1^H-NMR (300 MHz, DMSO-*d*_6_): δ 10.70 (s, 1H, H9), 8.67 (s, 1H, H5′), 8.33 (s, 1H, H5″), 7.96 (d, *J* = 8.55 Hz, 1H, H7″), 7.88 (d, *J* = 8.95 Hz, 2H, H3″, H8″), 7.34–7.32 (m, 2H, H1″, H5), 7.26 (d, *J* = 7.85 Hz, 1H, H8), 7.18 (dd, *J* = 8.94, 2.44 Hz, 1H, H3″), 6.99 (t, *J* = 7.49, 7.49 Hz, 1H, H7), 6.91 (t, *J* = 6.94, 6.94 Hz, 1H, H6), 4.58 (s, 2H, H10), 3.94 (s, 2H, H1), 3.88 (s, 3H, CH_3_), 2.67 (d, *J* = 12.69 Hz, 1H, H4_b_), 2.49 (m, 1H, H4_a_); ^13^C-NMR (300 MHz, DMSO-*d*_6_): δ 157.530, 146.446, 135.899, 133.942, 129.594, 128.655, 127.447, 127.097, 126.202, 124.242, 123.485, 122.075, 120.479, 119.159, 118.358, 117.193, 110.949, 106.168, 106.014, 55.310, 54.164, 53.498, 41.959, 25.484; LRMS (API-ES) *m/z* 841.4 [2M+Na], 432.1 [M+Na], 410.3 [M+H]; HRMS (ESI) *m/z* calcd for [M^+^] 409.4830, Found 432.1699 [M+Na], 401.1896 [M+H]. 

*(S)-3-((4-(3,4-Dichlorophenyl)-1H-1,2,3-triazol-1-yl)methyl)-2,3,4,9-tetrahydro-1H-pyrido[3,4-b]- indole* (**6c**). Compound **6c** was obtained from compound **5** and 1,2-dichloro-4-ethynylbenzene (**c**) as described above. The concentrated residue was purified by column chromatography (CHCl_3_/ EtOAc/NH_4_OH; 5:5:0.1). A light orange powder of compound **6c** was obtained (0.2142 g, 52%); m.p.: 220–221 °C; FTIR (KBr): 3292, 1554, 1130, 803 cm^−1^; ^1^H-NMR (300 MHz, DMSO-*d*_6_): δ 10.71 (s, 1H, H9), 8.75 (s, 1H, H5′), 8.11 (d, *J* = 1.93 Hz, 1H, H2″), 7.86 (dd, *J* = 8.39, 1.98 Hz, 1H, H6″), 7.70 (dd, *J* = 8.41, 1.90 Hz, 1H, H5″), 7.33 (d, *J* = 7.56 Hz, 1H, H5), 7.26 (d, *J* = 7.8 Hz, 1H, H8), 6.99 (t, *J* = 7.49 Hz, 1H, H7), 6.91 (t, *J* = 7.64 Hz, 1H, H6), 4.63–4.49 (m, 2H, H10), 3.94 (d, *J* = 16.66 Hz, 1H, H1_b_), 3.87 (d, *J* = 16.73 Hz, 1H, H1_a_), 3.32 (m, 1H, H3), 2.68 (dd, *J* = 14.85, 3.63 Hz, 1H, H4_b_), 2.50–2.41 (m, 1H, H4_a_); ^13^C-NMR (300 MHz, DMSO-*d*_6_): δ 143.870, 135.752, 133.878, 131.684, 131.574, 131.115, 129.945, 126.969, 126.679, 125.099, 123.058, 120.320, 118.190, 117.059, 110.806, 105.850, 54.129, 53.325, 41.821, 25.357; LRMS (API-ES) *m/z* 420.2 [M+Na], 398.2 [M^+^]; HRMS (ESI) *m/z* calcd for [M^+^] 398.2885, Found 420.0629 [M+Na]. 

*(S)-3-((4((N-(3-(4-Hydroxybenzoyl)benzoyl)azyl)methyl)-1H-1,2,3-triazol-1-yl)methyl)-2,3,4,9-tetra-hydro-1H-pyrido[3,4b]indole* (**6d**). Compound **6d** was obtained from compound **5** and 2-(4-hydroxybenzoyl)-*N*-(prop-2-ynyl)benzamide (**d**) as described above. The concentrated residue was purified by column chromatography (EtOAc/EtOH/NH_4_OH; 9:1:0.1). A light brown powder of compound **6d** was obtained (0.1773 g, 35%); m.p.: 213–215 °C; FTIR (KBr): 3409, 1682, 1613, 1513, 1372, 1277, 1169 cm^−^^1^; ^1^H-NMR (400 MHz, DMSO-*d*_6_): δ 10.67 (s, 1H, H1), 9.47 (s, 1H, OH), 7.75 (d, *J* = 5.2 Hz, 1H, H6″), 7.71 (d, *J* = 7.2 Hz, 1H, H5″), 7.56–7.47 (m, 2H, H4″, H5″), 7.30 (t, *J* = 6.80 Hz, 1H, H7′), 7.24 (d, *J* = 7.60 Hz, 2H, H5, H8), 7.09 (d, *J* = 8.4 Hz, 2H, H2″′, H6″′), 7.00–6.89 (m, 3H, H7, H5′, H6), 6.65 (dd, *J* = 8.60, 2.60 Hz, 2H, H3″′, H5″′), 4.59 (d, *J* = 15.2 Hz, H6′_b_), 4.41–4.34 (m, 2H, H10), 4.22 (dd, *J* = 15.8, 3.4 Hz, 1H, H6′_a_), 3.91 (d, *J* = 16.0 Hz, 1H, H1_b_), 3.84 (d, *J* = 16.4 Hz, 1H, H1_a_), 3.25–3.23 (m, 1H, H3), 2.60–2.55 (m, 1H, H4_a_), 2.38–2.31 (m, 1H, H4_b_); ^13^C-NMR (300 MHz, DMSO-*d*_6_): δ 166.605, 157.196, 150.040, 135.937, 132.581, 130.104, 129.597, 129.032, 127.317, 126.899, 122.803, 122.470, 120.561, 118.344, 117.186, 115.036, 110.939, 90.755, 59.783, 34.500; LRMS (ESI) *m/z* 529.27 [M+Na], 489.12 [M^+^-17]; HRMS (ESI) *m/z* calcd for [M^+^] 506.5551, Found 529.1922[M+Na], 489.1922 [M^+^-17].

*(S)-3-((4-(N-(((4-Hydroxyphenyl)propanoyl)azyl)methyl)-1H-1,2,3-triazol-1-yl)methyl)-2,3,4,9-tetra- hydro-1H-pyrido[3,4-b]ind ole* (**6e**). Compound **6e** was obtained from compound **5** and 3-(4-hydroxyphenyl)-*N*-(prop-2-ynyl)propanamide (**e**) as described above. The concentrated residue was washed with chloroform, ethyl acetate (20 mL) for each solvent. A yellow brown powder of compound **6e** was obtained (0.1685 g, 39%); m.p.: 218–220 °C; FTIR (KBr): 3407, 3303, 3262, 1641, 1514, 1381, 1261, 1153 cm^−^^1^; ^1^H-NMR (300 MHz, DMSO-*d*_6_): δ 10.77 (s, ,1H, H9), 9.21 (s, 1H, OH), 8.35 (t, *J* = 5.30 Hz, 1H, H7′), 7.78 (s, 1H, H5′), 7.31 (d, *J* = 7.65 Hz, 1H, H5), 7.26 (d, *J* = 7.90 Hz, 1H, H8), 7.02–6.89 (m, 4H, H7, H2″, H6″, H6), 6.65 (d, *J* = 8.29 Hz, 2H, H3″, H5), 4.52 (s, 1H, H10), 4.28 (d, *J* = 5.39 Hz, 2H, H1), 3.96 (s, 2H, H6′), 2.70 (t, *J* = 7.63 Hz, 2H, H9′), 2.63 (br, 1H, H4_b_), 2.43 (br, 1H, H4_a_) 2.34 (t, *J* = 7.71 Hz, 2H, H10′); ^13^C-NMR (300 MHz, DMSO-*d*_6_): δ 171.55, 155.479, 135.881, 131.295, 129.125, 126.844, 120.572, 118.377, 117.179, 115.071, 110.980, 105.700, 53.304, 37.338, 34.233, 30.332; LRMS (ESI) *m/z* 453.60 [M+Na]; HRMS (ESI) *m/z* calcd for [M^+^] 430.5022, Found 453.1911 [M+Na]. 

*(S)-3-((4-(((N-(3,5-Dihydroxy)benzoyl)azyl)methyl)-1H-1,2,3-triazol-1-yl)methyl)-2,3,4,9-tetrahydro-1H-pyrido[3,4-b]indole*(**6f**). Compound **6f** was obtained from compound **5** and 3,5-dihydroxy-*N*-(prop-2-ynyl)benzamide (**f**) as described above. The concentrated residue was purified by column chromatography (EtOAc/EtOH/NH_4_OH; 10:1:0.1). A light pink powder of **6f** was obtained (0.0937 g, 22%); m.p.: 180–182 °C; FTIR (KBr): 3395, 3284, 1644, 1596, 1543, 1353, 1163, 1004 cm^−1^; ^1^H-NMR (300 MHz, DMSO-*d*_6_): δ 10.71 (s, 1H, H9), 9.48 (s, 2H, OH), 8.79 (t, *J* = 5.61 Hz, 1H, H7′), 7.98 (s, 1H, H5′), 7.30 (d, *J* = 7.58 Hz, 1H, H5), 7.25 (d, *J* = 7.85 Hz, 1H, H8), 6.99 (t, *J* = 6.94 Hz, 1H, H7), 6.92 (t, *J* = 7.34 Hz, 1H, H6), 6.71 (d, *J* = 2.13 Hz, 2H, H2″, H6″), 6.37 (d, *J* = 2.10 Hz, 1H, H4″), 4.56–4.43 (m, 4H, H10, H1), 4.05–3.85 (m, 2H, H6′), 3.34 (m, 1H, H3), 2.62 (dd, *J* = 15.02, 3.46 Hz, 1H, H4_b_), 2.39 (dd, *J* = 14.62, 9.51 Hz, 1H, H4_a_); ^13^C-NMR (300 MHz, DMSO-*d*_6_): δ 166.446, 158.288, 145.029, 136.437, 135.830, 133.438, 126.934, 123.752, 120.481, 118.345, 117.152, 110.921, 105.846, 105.568, 105.219, 53.574, 53.513, 41.831, 34.923, 25.176; LRMS (ESI) *m/z* 441.27 [M+Na], 419.14 [M+H]; HRMS (ESI) *m/z* calcd for [M^+^] 418.4485, Found 419.2116 [M+H]. 

*(S)-3-((4-(N-(((E)-(3-Methoxy-4-hydroxyphenyl)propenoyl)azyl)methyl)-1H-1,2,3-triazol-1-yl)-methyl)-2,3,4,9-tetrahydro-1H-pyrido[3,4-b]indole* (**6g**). Compound **6g** was obtained from compound **5** and(*E*)-3-(4-hydroxy-3-methoxyphenyl)-*N*-(prop-2-ynyl)acrylamide (**g**) as described above. The concentrated residue was purified by column chromatography (EtOAc/EtOH/NH_4_OH; 9:1:0.1). A light yellow powder of **6g** was obtained (0.1356 g, 30%); m.p.: 199–200 °C; FTIR (KBr): 3373, 3262, 1650, 1584, 1517, 1327, 1280, 1204, 1169, 1030 cm^−^^1^;^ 1^H-NMR (300 MHz, DMSO-*d*_6_): δ 10.69 (s, 1H, H9), 9.45 (s, 1H, OH), 8.46 (s, 1H, H7′), 8.01 (s, 1H, H5′), 7.40–7.24 (m, 3H, H10′, H5, H8), 7.12 (s, 1H, H2″), 7.01–6.98 (m, 2H, H7, H6″), 6.91 (t, *J* = 7.34 Hz, 1H, H6), 6.79 (d, *J* = 8.14 Hz, 1H, H5″), 6.50 (d, *J* = 15.70 Hz, 1H, H9′), 4.49–4.44 (m, 4H, H10, H1), 3.93–3.87 (m, 2H, H6′), 3.79 (s, 3H, CH_3_), 3.29 (s, 1H, H3), 2.63–2.34 (m, 2H, H4); ^13^C-NMR (300 MHz, DMSO-*d*_6_): δ 165.330, 148.322, 147.807, 144.567, 139.392, 135.766, 133.904, 126.981, 126.351, 123.649, 121.507, 120.370, 118.675, 118.246, 117.081, 115.674, 110.859, 105.916, 55.512, 53.806, 53.506, 41.891, 34.383, 25.370; LRMS (ESI) *m/z* 459.42 [M+H]; HRMS (ESI) *m/z* calcd for [M^+^] 458.5123, Found 481.1977 [M+Na], 459.2158 [M+H].

*(S)-3-((4-(N-(((E)-(3,4-Dihydroxyphenyl)propenoyl)azyl)methyl)-1H-1,2,3-triazol-1-yl)methyl)-2,3,4,9-tetrahydro-1H-pyrido[3,4-b]indole* (**6h**). Compound **6h** was obtained from compound **5** and((*E*)-3-(3,4-dihydroxyphenyl)-*N*-(prop-2-ynyl)acrylamide (**h**) as described above. The concentrated residue was purified by column chromatography (EtOAc/EtOH/NH_4_OH; 9:1:0.1). A light yellow powder of **6h** was obtained (0.0710 g, 18%); m.p.: 176–178 °C; FTIR (KBr): 3392, 3271, 1650, 1600, 1527, 1375, 1283, 1112 cm^−^^1^; ^1^H-NMR (300 MHz, DMSO-*d*_6_): δ 10.76 (s, 1H, H9), 9.20 (m, 2H, OH), 8.54 (s, 1H, H7′), 8.04 (s, 1H, H5′), 7.35–7.27 (m, 3H, H5, H8, H10′), 7.04–6.91 (m, 3H, H7, H6, H2″), 6.85 (d, *J* = 8.24 Hz, 1H, H6″), 6.76 (d, *J* = 8.03 Hz, 1H, H5″), 6.40 (d, *J* = 15.72 Hz, 1H, H9′), 4.55–4.44 (m, 4H, H10, H1), 4.03–3.42 (m, 2H, H6′), 3.48–3.41 (m, 1H, H3), 2.67 (d, *J* = 11.88 Hz, 1H, H4_b_), 2.51–2.45 (m, 1H, H4_a_); ^13^C-NMR (300 MHz, DMSO-*d*_6_): δ 165.369, 147.396, 145.552, 144.686, 139.542, 135.830, 132.971, 126.831, 126.305, 123.783, 120.523, 118.354, 118.152, 117.158, 115.770, 113.801, 110.933, 105.716, 53.439, 53.342, 41.738, 34.334, 24.949; LRMS (ESI) *m/z* 445.50 [M+H]; HRMS (ESI) *m/z* calcd for [M^+^] 444.4858, Found 467.1815 [M+Na], 445.2002 [M+H]. 

*(S)-1-(4-(4-tert-Butylphenyl)-1H-1,2,3-triazol-1-yl)-3-(1H-indol-3-yl)propan-2-amine* (**12a**). Compound **12a** was obtained from compound **11** and 1-*tert*-butyl-4-ethynylbenzene (**a**) as described above. The concentrated residue was purified by column chromatography (CHCl_3_/EtOAc/NH_4_OH; 9:1:0.1) to yield a brown semisolid of compound **12a** (0.1530 g, 41%); FTIR (ATR): 3407, 3358, 3287, 1665, 1583, 1265, 1051 cm^−1^; ^1^H-NMR (300 MHz, DMSO-*d*_6_): δ 10.89 (s, 1H, H1), 8.49 (s, 1H, H5′), 7.75 (d, *J* = 8.36 Hz, 2H, H2″, H6″), 7.52 (d, *J* = 7.80 Hz, 1H, H4), 7.44 (d, *J* = 8.40 Hz, 2H, H3″, H5″), 7.35 (d, *J* = 8.02 Hz, 1H, H7), 7.23 (d, *J* = 2.10 Hz, 1H, H2), 7.06 (t, *J* = 7.09 Hz, 1H, H6), 6.97 (t, *J* = 7.39 Hz, 1H, H5), 4.41 (dd, *J* = 13.46, 4.56 Hz, 1H, H10_b_), 4.23 (dd, *J* = 13.54, 7.76 Hz, 1H, H10_a_), 3.40 (m, 1H, H9), 2.81 (dd, *J* = 14.22, 5.80 Hz, 1H, H8_b_), 2.69 (dd, *J* = 14.22, 7.13 Hz, 1H, H8_a_), 1.29 (s, 9H, CH_3_); ^13^C-NMR (300 MHz, DMSO-*d*_6_): δ 150.171, 145.965, 136.265, 128.177, 127.446, 125.561, 124.900, 123.718, 121.816, 120.917, 118.360, 118.304, 111.409, 110.646, 55.733, 52.042, 34.321, 31.060; LRMS (API-ES) *m/z* 374.4 [M+H]; HRMS (ESI) *m/z* calcd for [M^+^] 373.4940, Found 374.2425 [M+H].

*(S)-1-(1H-Indol-3-yl)-3-(4-(6-methoxynaphthalen-2-yl)-1H-1,2,3-triazol-1-yl)propan-2-amine* (**12b**). Compound **12b** was obtained from compound **11** and 2-ethynyl-6-methoxynaphthalene (**b**) as described above. The concentrated residue was purified with column chromatography (CHCl_3_/EtOAc/NH_4_OH; 8:2:0.1) to yield a white solid of **12b** (0.1078 g, 27%); m.p.: 85–87 °C; FTIR (KBr): 3413, 3274, 1612, 1549, 1260, 1163, 1030 cm^−1^; ^1^H-NMR (400 MHz, DMSO-*d*_6_): δ 10.89 (s, 1H, H9), 8.60 (s, 1H, H5′), 8.30 (s, 1H, H5″), 7.93 (dd, *J* = 8.52, 1.55 Hz, 1H, H7″), 7.86 (d, *J* = 9.29 Hz, 2H, H4″, H8″), 7.53 (d, *J* = 7.86 Hz, 1H, H4), 7.34 (d, *J* = 8.07 Hz, 1H, H7), 7.31 (d, *J* = 2.40 Hz, 1H, H1″), 7.23 (d, *J* = 2.17 Hz, 1H, H5″), 7.16 (dd, *J* = 8.95, 2.51 Hz, 1H, H3″), 7.05 (t, *J* = 7.52 Hz, 1H, H6), 6.96 (t, *J* = 7.44 Hz, 1H, H5), 4.43 (dd, *J* = 13.53, 4.53 Hz, 1H, H10_b_), 4.25 (dd, *J* = 13.52, 7.83 Hz, 1H, H10_a_), 3.47–3.41 (m, 1H, H9), 2.82 (dd, *J* = 14.22, 5.89 Hz, 1H, H8_a_), 2.70 (dd, 14.24, 7.13 Hz, 1H, H8_b_), 1.56 (s, 2H, NH_2_); ^13^C-NMR (300 MHz, DMSO-*d*_6_): δ 157.407, 146.277, 136.297, 133.824, 129.491, 128.547, 127.312, 126.121, 124.137, 123.925, 123.352, 122.189, 120.989, 119.091, 118.375, 111.461, 110.084, 106.019, 55.210, 54.893, 51.857, 30.201; LRMS (API-ES) *m/z* 817.5 [2M+Na], 398.4 [M+H]; HRMS (ESI) *m/z* calcd for [M^+^] 397.4723, Found 398.2066 [M+H]. 

*(S)-1-(4-(3,4-Dichlorophenyl)-1H-1,2,3-triazol-1-yl)-3-(1H-indol-3-yl)propan-2-amine* (**12c**). Compound **12c** was obtained from compound **11** and 1,2-dichloro-4-ethynylbenzene (**c**) as described above. The concentrated residue was purified with column chromatography (CHCl_3_/EtOAc/NH_4_OH; 9:1:0.1) to yield a light pink powder of compound **12c** (0.2500 g, 65%); m.p.: 149–151 °C; FTIR (KBr): 3446, 3357, 3264, 1608, 1555, 1132, 800 cm^−1^; ^1^H-NMR (300 MHz, DMSO-*d*_6_): δ 10.89 (s, 1H, H1), 8.68 (s, 1H, H5′), 8.08 (d, *J* = 1.86 Hz, 1H, H2″), 7.84 (dd, *J* = 8.39, 1.94 Hz, 1H, H6″), 7.69 (d, *J* = 8.4 Hz, 1H, H5″), 7.53 (d, *J* = 7.78 Hz, 1H, H4), 7.35 (d, *J* = 7.97 Hz, 1H, H7), 7.23 (d, *J* = 1.97 Hz, 1H, H2), 7.06 (t, *J* = 7.48 Hz, 1H, H6), 6.97 (t, *J* = 7.38 Hz, 1H, H5), 4.43 (dd, *J* = 13.52, 4.38 Hz, 1H, H10_b_), 4.24 (dd, *J* = 13.49, 7.88 Hz, 1H, H10_a_), 3.42 (m, 1H, H9), 2.82 (dd, *J* = 14.26, 5.92 Hz, 1H, H8_b_), 2.70 (dd, *J* = 14.25, 6.99 Hz, 1H, H8_a_), 1.54 (s, 2H, NH_2_); ^13^C-NMR (300 MHz, DMSO-*d*_6_): δ 143.770, 136.265, 131.680, 131.136, 129.909, 127.427, 126.682, 125.096, 123.713, 123.186, 120.924, 118.363, 118.310, 111.412, 110.631, 55.942, 52.023, 31.081; LRMS (API-ES) *m/z* 386.2 [M^+^]; HRMS (ESI) *m/z* calcd for [M^+^] 386.2778, Found 386.1003 [M^+^]. 

*(S)-N-((1-(2-Amino-3-(1H-indol-3-yl)propyl)-1H-1,2,3-triazol-4-yl)methyl)-2-(4-hydroxybenzoyl)-benzamide *(**12d**). Compound **12d** was obtained from compound **11** and 2-(4-hydroxybenzoyl)-N-(prop-2-ynyl)benzamide (**d**) as described above. The concentrated residue was purified with column chromatography (EtOAc/EtOH/NH_4_OH; 9:1:0.1) to yield a light yellow powder of compound **12d** (0.1086 g, 22%); m.p.: 177–179 °C; FTIR (KBr): 3404, 3347,1684, 1612, 1511, 1394, 1202, 1052 cm^−1^; ^1^H-NMR (400 MHz, DMSO-*d*_6_): δ 10.98 (s, 1H, H1), 7.78 (s, 1H, H5′), 7.77 (s, 1H, H6″), 7.62–7.53 (m, 3H, H4, H4″, H5″), 7.40 (d, *J* = 8.00 Hz, 1H, H7), 7.30 (d, *J* = 7.20 Hz, 1H, H3″), 7.25 (d, *J* = 1.60 Hz, 1H, H2), 7.13 (d, *J* = 8.40 Hz, 2H, H2″′, H6″′), 7.10 (d, *J* = 7.6 Hz, 1H, H6), 7.01 (t, *J* = 7.40 Hz, 1H, H5), 6.71 (d, *J* = 8.4 Hz, 2H, H3″′, H5″′), 4.64 (d, *J* = 15.60 Hz, 1H, H6′_b_), 4.34–4.29 (m, 2H, H10), 4.26 (d, *J* = 15.6 Hz, H6′_a_) 4.18–4.22 (m, 1H, H10), 2.81–2.76 (m, 1H, H8_a_), 2.60 (dd, *J* = 14.00, 7.20 Hz, 1H, H8_b_); ^13^C-NMR (400 MHz, DMSO-*d*_6_): δ 166.999, 157.809, 150.502, 144.332, 136.705, 132.968, 130.543, 129.864, 129.422, 127.881, 127.675, 124.327, 124.121, 123.221, 122.885, 121.337, 118.836, 118.736, 115.503, 118.842, 111.125, 91.188, 55.899, 52.452, 34.719, 31.257; LRMS (API-ES) *m/z* 1011.3 [2M+Na], 477.2 [M^+^-17]; HRMS (ESI) *m/z* calcd for [M^+^] 494.5444, Found 477.1888 [M^+^-17]. 

*(S)-N-((1-(2-Amino-3-(1H-indol-3-yl)propyl)-1H-1,2,3-triazol-4-yl)methyl)-3-(4-hydroxyphenyl)propanamide* (**12e**). Compound **12e** was obtained from compound **11** and 3-(4-hydroxyphenyl)-*N*-(prop-2-ynyl)propanamide (**e**) as described above. The concentrated residue was purified with column chromatography (CHCl_3_/MeOH/NH_4_OH; 10:0.2:0.1) to yield brown semisolid of compound **12e** (0.2007 g, 49%): FTIR (ATR): 3398, 3338, 3281, 1650, 1613, 1515, 1340, 1232, 1052 cm^−1^; ^1^H-NMR (300 MHz, DMSO-*d*_6_): δ 10.90 (s, 1H, H1), 8.31 (t, *J* = 5.50 Hz, 1H, H7′), 7.74 (s, 1H, H5′), 7.52 (d, *J* = 7.79 Hz, 1H, H4), 7.36 (d, *J* = 8.00 Hz, 1H, H7), 7.23 (s, 1H, H2), 7.08 (t, *J* = 7.13 Hz, 1H, H6), 7.01–6.96 (m, 3H, H5, H2″, H6″), 6.66 (d, *J* = 8.37 Hz, 2H, H3″, H5″), 4.38–4.14 (m, 4H, H10, H6′), 3.34 (s, 1H, H9), 2.81–2.62 (m, 4H, H8, H9′), 2.35 (t, *J* = 7.75 Hz, 2H, H10′); ^13^C-NMR (300 MHz, DMSO-*d*_6_): δ 171.437, 155.429, 144.662, 136.286, 131.321, 129.111, 127.425, 123.704, 123.565, 120.951, 118.376, 118.334, 115.040, 111.433, 110.645, 55.350, 52.098, 37.310, 34.205, 30.923, 30.287; LRMS (API-ES) *m/z* 859.5 [2M+Na], 441.4 [M+Na], 419.3 [M+H]; HRMS (ESI) *m/z* calcd. for [M^+^] 418.4915, Found 441.1939 [M+Na].

*(S)-N-((1-(2-Amino-3-(1H-indol-3-yl)propyl)-1H-1,2,3-triazol-4-yl)methyl)-3,5-dihydroxybenzamide* (**12f**). Compound **12f** was obtained from compound **11** and 3,5-dihydroxy-*N*-(prop-2-ynyl) benzamide (**f**) as described above. The concentrated residue was purified with column chromatography (CHCl_3_/MeOH/NH_4_OH; 10:0.4:0.1) to yield compound **12f** as a light orange solid (0.1304 g, 32%); m.p.: 68–70 °C; FTIR (KBr): 3415, 1679, 1595, 1536, 1340, 1206, 1165 cm^−1^; ^1^H-NMR (300 MHz, DMSO-*d*_6_): δ 10.91 (s, 1H, H1), 9.51 (s, 2H, OH), 8.76 (t, *J* = 5.58 Hz, 1H, H7′), 7.95 (s, 1H, H5′), 7.50 (d, *J* = 7.74 Hz, 1H, H4), 7.35 (d, *J* = 8.02 Hz, 1H, H7), 7.22 (d, *J* = 2.00 Hz, 1H, H2), 7.07 (t, *J* = 7.09 Hz, 1H, H6), 6.96 (t, *J* = 7.05 Hz, 1H, H5), 6.71 (d, *J* = 2.08 Hz, 2H, H2″, H6″), 6.73 (s, 1H, H4″), 4.46 (d, *J* = 5.55 Hz, 2H, H6′), 4.37 (dd, *J* = 13.55, 4.41 Hz, 1H, H10_b_), 4.20 (dd, *J* = 13.48, 7.66 Hz, 1H, H10_a_), 3.34 (m, 1H, H9), 2.77 (dd, *J* = 14.41, 5.94 Hz, 1H, H8_b_), 2.66 (dd, *J* = 14.15, 7.30 Hz, 1H, H8_a_); ^13^C-NMR (300 MHz, DMSO-*d*_6_): δ 166.409, 158.268, 144.853, 136.426, 136.289, 127.411, 123.799, 123.733, 120.948, 118.350, 111.439, 110.568, 105.537, 105.179, 55.221, 52.074, 34.907, 30.829; LRMS (API-ES) *m/z* 429.3 [M+Na], 407.5 [M+H]; HRMS (ESI) *m/z* calcd for [M^+^] 406.4378, Found 429.1615 [M+Na], 407.1798 [M+H]. 

*(S,E)-N-((1-(2-Amino-3-(1H-indol-3-yl)propyl)-1H-1,2,3-triazol-4-yl)methyl)-3-(4-hydroxy-3-methoxyphenyl)acrylamide *(**12g**). Compound **12g** was obtained from compound **11** and (*E*)-3-(4-hydroxy-3-methoxyphenyl)-*N*-(prop-2-ynyl)acrylamide (**g**) as described above. The concentrated residue was purified by column chromatography (CH_2_Cl_2_/EtOH/NH_4_OH; 9.5:0.5:0.1) to yield compound **12g** as a yellow solid (0.1020 g, 23%); m.p.: 118–119 °C; FTIR (KBr): 3395, 3344, 3262, 1656, 1593, 1511, 1336, 1277, 1229, 1121, 1030 cm^−1^; ^1^H-NMR (300 MHz, DMSO-*d*_6_): δ 10.88 (s, 1H, H1), 8.43 (t, *J* = 5.68 Hz, 1H, H7′), 7.98 (s, 1H, H5′), 7.51 (d, *J* = 9.73 Hz, 1H, H4), 7.39–7.34 (m, 2H, H10′, H7), 7.22 (d, *J* = 2.13 Hz, 1H, H2), 7.12 (d, *J* = 1.69 Hz, H2″), 7.06 (t, *J* = 7.49 Hz, 1H, H6), 7.02–6.94 (m, 2H, H6″, H5), 6.79 (d, *J* = 8.11 Hz, 1H, H5″), 6.49 (d, *J* = 15.69 Hz, 1H, H9′), 4.43 (d, *J* = 5.47 Hz, 2H, H6′), 4.36 (dd, *J* = 13.44, 4.60 Hz, 1H, H10_b_), 4.19 (dd, *J* = 13.42, 7.56 Hz, 1H, H10_a_), 3.80 (s, 3H, CH_3_), 3.36–3.32 (m, 1H, H9), 2.77 (dd, *J* = 14.27, 5.81 Hz, 1H, H8_b_), 2.65 (dd, *J* = 14.18, 7.23 Hz, 1H, H8_a_); ^13^C-NMR (300 MHz, DMSO-*d*_6_): δ 165.283, 148.304, 147.797, 144.437, 139.345, 136.257, 127.405, 126.336, 123.705, 123.664, 121.496, 120.910, 118.661, 118.352, 118.308, 115.653, 111.399, 110.838, 110.680, 55.509, 55.409, 52.102, 34.372, 31.018; LRMS (ESI) *m/z* 469.58 [M+Na], 447.58 [M+H]; HRMS (ESI) *m/z* calcd for [M^+^] 446.5016, Found 469.1923 [M+Na], 447.2119 [M+H].

*(S,E)-N-((1-(2-Amino-3-(1H-indol-3-yl)propyl)-1H-1,2,3-triazol-4-yl)methyl)-3-(3,4-dihydroxy- phenyl)acrylamide* (**12h**). Compound **12h** was obtained from compound **11** and ((*E*)-3-(3,4-dihydroxyphenyl)-*N*-(prop-2-ynyl)acrylamide (**h**) as described above. The concentrated residue was purified by column chromatography (CHCl_3_/EtOH; 8:2) to compound **12h** as a yellow brown semisolid (0.0562 g, 13%): FTIR (ATR): 3404, 3284, 1656, 1593, 1524, 1340, 1270, 1118 cm^−1^; ^1^H-NMR (400 MHz, DMSO-*d*_6_): δ 10.92 (s, 1H, H1), 8.51–8.46 (m, 1H, H7′), 7.96 (s, 1H, H5′), 7.49 (d, *J* = 7.60 Hz, 1H, H4), 7.33 (d, *J* = 8.40 Hz, 1H, H10′), 7.27 (s, 1H, H2), 7.23–7.19 (m, 2H, H7, H5″), 7.05 (t, *J* = 7.60 Hz, 1H, H6), 6.96 (t, *J* = 7.40 Hz, 1H, H5), 6.92 (d, *J* = 2.00 Hz, 1H, H2″), 6.81 (dd, *J* = 8.40, 2.00 Hz, 1H, H6″), 6.72 (d, *J* = 8.40 Hz, 1H, H9′), 4.40–4.36 (m, 3H, H6′, H10_b_), 4.24 (dd, *J* = 13.80, 7.40 Hz, 1H, H10_a_), 2.78 (dd, *J* = 14.40, 6.40 Hz, 1H, H8_b_), 2.69 (dd, *J* = 14.20, 7.00 Hz, 1H, H8_a_); ^13^C-NMR (400 MHz, DMSO-*d*_6_): δ 171.956, 165.794, 147.848, 146.002, 145.034, 139.970, 136.721, 129.338, 127.782, 126.729, 124.289, 121.413, 120.903, 118.813, 118.584, 116.204, 114.267, 111.880, 52.315, 34.765, 22.928, 21.632; LRMS (ESI) *m/z* 455.67 [M+Na]; HRMS (ESI) *m/z* calcd for [M^+^] 432.4751, Found 455.1807 [M+Na], 447.2119 [M+H].

### 3.7. β-Secretase inhibition assay

β-Secretase enzyme was reconstituted with 50 mM Tris (pH 7.5), 10% glycerol to yield concentration of 0.5 unit/µL. The enzyme stock solution was diluted with 100 mM sodium acetate buffer (pH 4.5) to 0.01 unit/µL. Thirty microliters of enzyme solution were added to each well that contained 20 µL of 5% DMSO of test compound. Then, fifty microliters of 50 µM β-secretase substrate IV from Calbiochem were added to the reaction plate. The fluorescence was measured at E_x_ = 380 nm and E_m_ = 510 nm by using SpectraMax Gemini EM^TM^ [29]. β-Secretase inhibitor IV was used as positive control. The assays were run in triplicate. The results of test compound 25 µM were analyzed. Compounds having inhibitory activity more than 50% were further evaluated for IC_50_ and data were analyzed via GrahPad Prism 4 in nonlinear regression curve fit.

### 3.8. Cathepsin D assay

Sensolyte^®^520 Cathepsin D assay kit was purchased from Anaspec. Forty microliters of cathepsin D (0.25 µg/mL in 5 mM DTT assay buffer) was added to a greiner black 96 well plate. Ten microliters of each test compound (1000 µM in 10% DMSO) were added to each well. After 10 min incubation at 37 °C, 50 µL of 0.01 mM cathepsin D substrate was added to each well. The assay reactions were incubated for 30 min at 37 °C. The fluorescence was measured at E_x_ = 490 nm and E_m_ = 520 nm by using SpectraMax Gemini EM^TM^. Pepstatin A was included as a positive control. The assays were run in triplicate. The data were analyzed.

### 3.9. Amyloid β Preparation and Aggregation Assay

Stock solution of amyloid-β (1-42) peptide (Anaspec; 1384 µM in 1% NH_4_OH) was diluted with 50 mM Tris buffer (pH 7.4) to yield 25 µM working solution. Nine microliters amyloid-β solution was added to a greiner transparent 96 well plate. One microliter of each test compounds (1,000 µM in DMSO) were added to each well and mixed gently by tapping. The final concentrations of each compound were 100 µM in 10% DMSO. The reaction plate was incubated in dark at 37 °C for 48 h. After incubation, 200 µL of 5 µM ThT (Sigma) in 50 mM Tris buffer (pH 7.4) was added to each well. The fluorescence was measured at E_x_ = 446 nm and E_m_ = 490 nm by using SpectraMax Gemini EM^TM^ [30]. The assays were run in triplicate. Curcumin was used as a positive control. The result data were analyzed and compounds having inhibition over 50% were evaluated for IC_50_ values and data were analyzed via GraphPad Prism 4 by nonlinear regression curve fit. 

### 3.10. Fe (II) Chelation Capcity Assay

An aqueous solution of 0.2 mM ferrous sulphate was prepared. Fifty microliters of this solution was added to 96 well plate, then 40 µL of 500 µM of test compounds in 50% DMSO were added to each well followed by DI water to adjust the volume to 150 µL. The mixtures were incubated at room temperature for 10 min. After incubation, 50 µL of 1 mM ferrozine (Sigma) was added to the reaction mixture. The absorbance was measured at 562 nm by using infinite 200 pro^TM^, Tecan [31,32]. EDTA was used as a positive control. Chelation capacity of each compounds were calculated. Compounds having chelating capacity over than 50% were evaluated for stoichiometric ratio of compound: metal. 

### 3.11. Free Radical Scavenging Assay

The test samples were prepared in 50% DMSO at 500 µM. Seventy microliters of DPPH solution (500 µM in methanol) was added to 96 well plate. The assay mixture was adjusted to 80 µL volume by methanol. After adjusting the volume, 20 µL of test compounds was added to each well. The reaction plate was incubated at room temperature in dark for 30 min. The absorbance was measured at 517 nm by using infinite 200 pro^TM^, Tecan [33]. Ascorbic acid was used as a positive control. Percent inhibition was calculated and compounds showing activity were evaluated for the IC_50_ values. 

### 3.12. Cell Culture and Cell Viability Assay by MTT method

The SH-SY5Y cells were cultured in a medium containing minimum essential medium (MEM): F-12 (1:1), 10% fetal bovine serum, MEM non-essential amino acids (0.5×), 0.5 mM sodium pyruvate and 100 units/ml of penicillin and 100 μg/mL of streptomycin. The cells were maintained under a humidified incubator with 5% CO_2_ in air at 37 °C.

SH-SY5Y cells were seeded in 96-well plates (2 × 10^4^ cells per well) for 24 h. After 24 h, cells were treated with 10 µM of test compounds for 2 h prior to exposure to 1 µM aggregated β-amyloid (1-42). The aggregated β-amyloid was prepared by incubating in a medium without serum, penicillin and streptomycin at 37 °C for 72 h. The cells treated with aggregated β-amyloid were incubated for 24 h, 15 µL of MTT reagent (5 mg/mL MTT in serum free medium containing 10 μM HEPES) was added to each well and incubated at 37 °C for 3 h. The medium was removed from each well. One hundred microlitres of 0.04 N HCl in isopropanol was added to each well to dissolve blue formazan crystals, which is the product of MTT catalyzed by mitochondrial dehydrogenase. The absorbance was measured at 570/630 nm using a Synergy^TM^ HT multi-detection microplate reader (Bio-Tek Instruments, Winooski, VT, USA) [34].

## 4. Conclusions

In this research, the tryptoline core compound previously reported as BACE1 inhibitor was modified *in silico* to possess multi-modes of action for the treatment of Alzheimer’s disease *i.e.*, anti-amyloid aggregation, metal chelating and radical scavenging action. The *in silico* designed compounds are tryptoline- and tryptamine-based BACE1 inhibitors containing additional moieties to exert multi-functionality. Among sixteen new compounds, the major action of compound **6h** were anti-Aβ aggregation and antioxidative action. Two compounds, **12c** and **12h**, were multifunctional compounds with three actions. Compound **12c **acted as a BACE1 inhibitor, anti-amyloid aggregation and metal chelator, while compound **12h** was an Aβ aggregation blocker, chelator and antioxidant. The IC_50_ values of compound **12c** against BACE1 and amyloid-β aggregation were 20.75 µM and 83.23 µM, while the IC_50_ values of compound **12h** against amyloid-β aggregation and antioxidant were 56.39 µM and 92.70 µM. Furthermore, these compounds acted as metal chelators with a stoichiometric ratio of ligand per metal 3:1. Despite the fact that the individual activities at each targets of compounds **6h**, **12c** and **12h** were rather weak (in the micromolar range), the neuroprotective effect against Aβ1-42 insult in SH-SY5Y cells of these multifunctional ligands were better than that of single targeted ligands *i.e.*, BACE1 inhibitor IV and comparable to the potent nanomolar curcumin. The results indicated the success of the multifunction strategy which suits the multi-pathogenesis of AD by reducing the neurotoxicity cascade from Aβ1-42.
